# Lipid Peroxidation in Obesity: Can Bariatric Surgery Help?

**DOI:** 10.3390/antiox11081537

**Published:** 2022-08-07

**Authors:** Ana Maria Soldo, Ivo Soldo, Andrija Karačić, Marcela Konjevod, Matea Nikolac Perkovic, Tanja Matijevic Glavan, Martina Luksic, Neven Žarković, Morana Jaganjac

**Affiliations:** 1 Department of Gastroenterology, General Hospital “Dr. Ivo Pedisic”, 44000 Sisak, Croatia; 2 Surgery Clinic, University Hospital Sveti Duh, 10000 Zagreb, Croatia; 3 Division of Molecular Medicine, Ruder Boskovic Institute, 10000 Zagreb, Croatia; 4 Department of Endocrinology, Diabetes and Metabolic Diseases, University Hospital Sveti Duh, 10000 Zagreb, Croatia

**Keywords:** obesity, adipose tissue dysfunction, redox homeostasis, lipid peroxidation, 4-hydroxynonenal, bariatric surgery

## Abstract

Obesity and chronic oxidative stress, often being associated with each other in a vicious circle, are important factors of chronic diseases. Although it was usually considered to accompany aging and wealth, global trends show the increase in obesity among children even in Third World countries. Being manifested by an imbalance between energy consumption and food intake, obesity is characterized by an excessive or abnormal fat accumulation, impaired redox homeostasis and metabolic changes often associated with the self-catalyzed lipid peroxidation generating 4-hydroxynonenal, pluripotent bioactive peroxidation product of polyunsaturated fatty acids. Conservative methods targeting obesity produced only modest and transient results in the treatment of morbid obesity. Therefore, in recent years, surgery, primarily bariatric, became an attractive treatment for morbid obesity. Since adipose tissue is well known as a stress organ with pronounced endocrine functions, surgery results in redox balance and metabolic improvement of the entire organism. The source of bioactive lipids and lipid-soluble antioxidants, and the complex pathophysiology of lipid peroxidation should thus be considered from the aspects of personalized and integrative biomedicine to treat obesity in an appropriate way.

## 1. Obesity

Obesity is a complex medical condition resulting from a chronic imbalance between energy consumption and food intake, and is diagnosed according to the body mass index (BMI) ≥ 30 kg/m^2^ [1,2]. Obesity is characterized by an excessive or abnormal fat accumulation and metabolic changes, and represents a global, public health, and socioeconomic problem [3,4]. Obesity is linked to an elevated risk of premature disability and death, and with a range of co-morbidities including dementia, depression, musculoskeletal and gallbladder disorders, metabolic syndrome, type 2 diabetes (T2DM), cardiometabolic diseases, and cancers [2,3,5,6]. The prevalence of worldwide obesity is increasing in all age groups, regardless of gender, and has tripled since mid-1970s [1,5,7]. The positive trend of increasing worldwide obesity is a result of various factors, including environmental, social, and individual factors, as well as food marketing. Other factors, including sleeping, certain medications, decreased smoking, global warming, ethnicity, and age, could also contribute to the expanse of worldwide obesity [8]. Today, behavioral therapy, pharmacotherapy and bariatric surgery are clinically significant and effective strategies for obese individuals [9]. However, to achieve and maintain an ideal weight, obese individuals require lifestyle changes such as increased physical activity, reduced calorie intake, and long-term treatment. The strategies to achieve this should be personalized according to other chronic disorders or preferences [10]. In this review, we discuss the consequences of obesity-induced oxidative stress and the impact of bariatric surgery.

## 2. Adipose Tissue Remodeling, Impairment of Redox Homeostasis and Dysfunction

**Figure 1:**
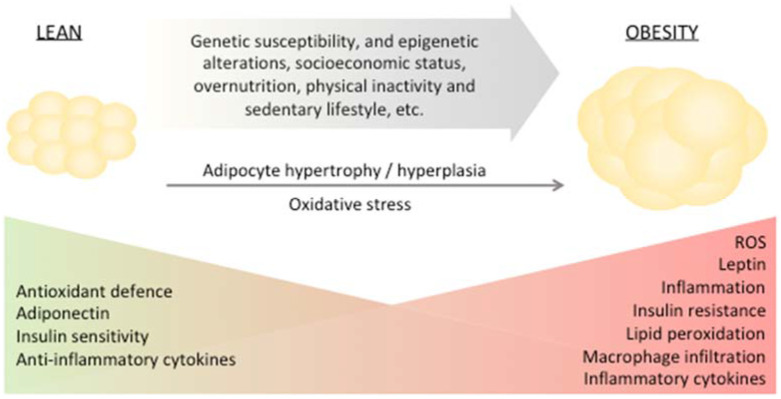
Genetic susceptibility and epigenetic alterations, socioeconomic status, overnutrition, physical inactivity and sedentary lifestyle are among the main factors driving the development of obesity. Obesity is marked by altered redox homeostasis in favor of prooxidants, inflammation, elevated adipose tissue macrophage infiltration and leptin secretion and decreased antioxidant response, insulin sensitivity and adiponectin. Eventually, these events result in oxidative stress and generation of lipid peroxidation-derived aldehydes promoting insulin resistance.

Adipose tissue is found in several depots within the body, each with a specific function [11]. In humans, the two major types of adipose tissue are white adipose tissue (WAT) and brown adipose tissue (BAT) [11]. WAT, predominantly composed of white adipocytes, represents the majority of human adipose tissue and has a substantial role in the control of body energy homeostasis. In response to excessive energy intake, in adults, WAT can expand by hypertrophy of present adipocytes or by increasing the number of adipocytes (hyperplasia) (Figure 1).

Among the major functions of WAT is the storage of triacylglycerols, and depending on the energy demands in order to maintain metabolic homeostasis, it releases fatty acids through lipolysis [11]. Contrary to WAT, the main function of BAT, composed mainly of brown adipocytes, is the expenditure of energy through non-shivering thermogenesis. Accordingly, white and brown adipocytes substantially differ in morphology and cellular processes. While white adipocytes have a large lipid droplet and only a few mitochondria, brown adipocytes have multiple smaller lipid droplets and high mitochondrial content that express uncoupling protein 1 (UCP1) [12]. The UCP1 drives the non-shivering thermogenesis in brown adipocytes [13]. BAT also has a role in adiposity and its metabolic activity is inversely related to BMI [14,15]. Although the prevalence of active BAT decreases with obesity [16], a recent study reported that obese individuals with active BAT have a healthier metabolic phenotype [17]. Besides white and brown adipocytes, today we also recognize beige, pink, and yellow adipocytes, all with distinct functions that are described elsewhere [18].

In addition to adipocyte fraction, the adipose tissue also contains the stromal vascular fraction (SVF). The heterogenous composition of the SVF fraction in response to altered metabolic demands will change over time. Among the most dynamic cells in SVF are macrophages [19]. In adipose tissue, there are two types of macrophages, type M1 macrophages associated with obesity and inflammation [20], and type M2 macrophages that secrete anti-inflammatory cytokines [21]. In proportion to the degree of adiposity, the number of macrophages rises from 5% of the cells to up to 50% of all cells in adipose tissue [22]. As their number increases, there is a shift in the M2/M1 balance in adipose tissue macrophages favoring inflammatory phenotype [23,24]. The cascade leading to adipose tissue dysfunction starts with accumulation of fat, followed by modifications in cellular composition, an increase in infiltrated inflammatory cells, and by the enlargement in adipocyte volume [25]. The above leads to increased secretion of a proinflammatory, atherogenic and diabetogenic adipokines as well as ROS which affects mRNA and protein expression patterns in adipose tissue cells [25]. Although low levels of ROS are essential for numerous cellular processes and functions and are essential to maintain the body’s homeostasis [26,27,28,29], high levels of ROS are harmful to the organism and contribute to the development and progression of numerous diseases [30]. Both enzymatic and non-enzymatic antioxidant defense mechanisms, help to maintain a healthy level of ROS. However, with age and with the duration of obesity, the effectiveness of antioxidant defense mechanisms declines, rendering cells more susceptible to ROS-induced damage [31]. When the amount of ROS generated overcomes the capacity of antioxidant defense systems, the redox homeostasis is disrupted, resulting in oxidative stress. Polyunsaturated fatty acids (PUFA) are particularly vulnerable to ROS at the bis-allylic site yielding reactive aldehydes, among which the bioactive 4-hydroxynonenal (4-HNE) is the most potent [32]. Indeed, elevated oxidative stress biomarkers, such as 4-HNE and altered antioxidant defense systems have been evidenced in obesity [33,34,35,36,37,38].

**Figure 2:**
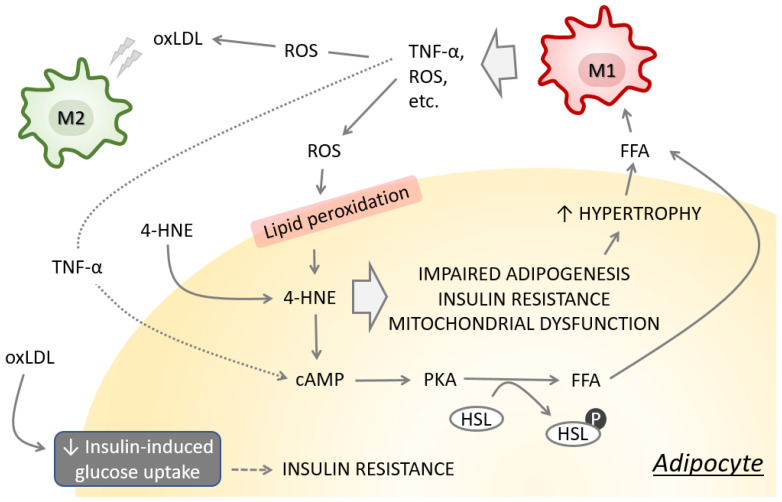
Altered redox homeostasis induces lipid peroxidation and promotes impaired adipogenesis, insulin resistance and mitochondrial dysfunction in adipocytes. These events consequently promote lipolysis and release of lipotoxic free fatty acids (FFA), promoting the TNF-α and ROS production by macrophages.

Interaction between macrophages and adipocytes is crucial in the initiation and maintenance of adipocyte dysfunction [39], and is, at least in part, attributed to intercellular redox signaling mediated by ROS and 4-HNE (Figure 2). We have shown that 4-HNE is responsible for impaired adipogenesis and induction of adipocyte insulin resistance [33,34]. Impaired adipogenesis may lead to adipocyte hypertrophy and, consequently, to hypoxic microenvironment, further promoting oxidative stress, adipose tissue inflammation and insulin resistance [40]. Enlarged adipocytes are responsible for increased secretion of free fatty acids that interact with macrophage Toll-like receptor-4 and activate transcription factor nuclear factor-κB (NF-κB), leading to enhanced tumor necrosis factor alpha (TNF-α) production [41,42] that eventually affects adipocytes by inducing lipolysis and altering gene expression [43,44]. Inflammation and oxidative stress affect lipid metabolism and triglyceride content in adipocytes by altering adipocyte gene expression, resulting in increased levels of circulating inflammatory cytokines and ROS which modulate systemic insulin action and substrate metabolism [45,46]. Oxidized low-density lipoprotein (oxLDL), associated with obesity and T2DM [47], affects the metabolism of visceral adipocytes toward insulin resistance phenotype [48]. Exposure of adipocytes to oxLDL further impairs the secretion of adipocytokines, affects cell death markers, induces NF-κB and Nrf2, and increases the expression of scavenger receptors [48]. M2 macrophages are particularly sensitive to oxLDL, which has lipotoxic effects by disturbing the function of the endoplasmic reticulum, initiating the unfolded protein response and promoting apoptosis [49], and they could contribute to the shift in the M2/M1 balance. The expression of adhesion molecules, such as intracellular adhesion molecule-1, and chemokines such as macrophage chemoattractant protein-1 by adipocytes further encourages diapedesis of blood monocytes to adipose tissue and their differentiation into macrophages [39]. In addition, adipokines, indirectly by activating NF-κB, enhance the NADPH oxidase activity and stimulate phagocytes, resulting in excessive superoxide radical production and oxidative stress. This vicious cycle keeps the macrophages and adipocytes in a persistent inflammatory state.

### Obesity—A Major Risk Factor for T2DM

Diabetes mellitus is marked by hyperglycemia, where fasting plasma glucose level is above ≥126 mg/dL, while levels between 99 mg/dL and 125 mg/dL are indicative of prediabetes. The major risk factors for the development of T2DM are aging, obesity and physical inactivity. Consuming foods high in calories leads to persistent hyperglycemia and hyperlipidemia, which over time worsen insulin sensitivity and secretion. Lipid droplets buffer excessive fatty acids on a cellular level by fat storage. However, once this capacity is exhausted, free fatty acids accumulate leading to lipotoxicity and promoting oxidative stress [50,51]. Moreover, early in T2DM development, visceral adipose tissue is the primary source of inflammation resulting from the response of resident adipose tissue macrophages to the changes in the microenvironment [19]. The progression of T2DM is accompanied by excessive macrophage infiltration into adipose tissue and the emergence of insulin resistance [1]. In addition to inflammation and lipotoxicity, hyperglycemia also induces oxidative stress and is one of the primary causes of impaired metabolic and endocrine homeostasis during T2DM pathogenesis [52]. Actually, hyperglycemia and impaired glucose metabolism precede the oxidation of fatty acids. Hyperglycemia promotes nonenzymatic glycation of proteins yielding advanced glycation end products (AGE). Binding of AGE to its receptor (RAGE), promotes intracellular ROS, activates NF-κB and inducible nitric oxide synthase (iNOS) [53,54]. Increased iNOS activity enhances the formation of the highly oxidizing peroxynitrite (ONOO^−^) as a result of the interaction between nitric oxide and superoxide. ONOO^−^ may either directly or indirectly affect different biomolecules, such as proteins, lipids and DNA, and thus impact various signaling pathways and cellular processes, as reviewed in [55]. Elevated levels of ONOO^−^ [31] were recently suggested to have a role in obesity-associated endothelial dysfunction by altering the cyclooxygenase pathway [56]. Both excessive ROS and RNS affect macromolecules and increase the risk of metabolic complications [31,32,34]. Peroxidation of lipids and the resulting 4-HNE directly correlates with glycated hemoglobin and fasting glucose in T2DM patients [52]. The relationship between obesity, reactive aldehydes, and pathology of diabetes is reviewed elsewhere [57]. In the section below, we discuss the consequences of obesity-induced lipid peroxidation, with the focus on 4-HNE.

## 3. Consequences of Obesity-Induced Lipid Peroxidation

Oxidative and nitrosative stress, and consequential lipid peroxidation have a critical role in the development of obesity and metabolic syndrome. Elevated 4-HNE was documented for both visceral and omental adipose tissue in obesity, and associated with adipose tissue dysfunction [33,34,58]. Furthermore, obesity-induced 4-HNE affects a plethora of processes such as ferroptosis, autophagy, proteostasis, adipogenesis, insulin signaling, and protein carbonylation (Table 1).

High-fat diet (HFD) promotes formation of 4-HNE and was found to promote ferroptosis and cardiac injury, and to affect autophagy and proteostasis [59,60]. Due to its high reactivity, 4-HNE readily forms adducts with proteins modulating various cellular processes and functions [32,57,61,62]. Indeed, elevated protein carbonylation in obesity is seen in both omental and subcutaneous adipose tissue of obese individuals, and was found to affect adipogenesis and promote insulin resistance [33,34]. Furthermore, in obesity, 4-HNE promotes secretion of adipokines [63] and forms adducts with a variety of proteins such as histones [64] and fatty acid binding protein [65]. An overview of different processes modulated by 4-HNE in obesity is given in Table 1.

**Table 1:** Processes affected by obesity-induced 4-HNE.

Process Affected	The Involvement of Obesity-Induced 4-HNE	Ref
Ferroptosis	HFD-induced obesity upregulates prostaglandin endoperoxide synthase 2 expression and promotes lipid peroxidation. Exosomes derived from obese adipose tissue macrophages upregulate prostaglandin endoperoxide synthase 2, promote formation of 4-HNE and induce mitochondrial injury. Obese adipose tissue macrophages exosomes contain a high level of miR-140-5p that affects GSH synthesis and promotes ferroptosis and cardiac injury in obesity.	[59]
Autophagy and proteostasis	HFD induces accumulation of lipid droplets in the liver and affects autophagy efficiency promoting accumulation of proteins modified with 4-HNE and 3-nitrotyrosine.	[60]
Carbonylation of histones	Obesity increases the level of 4-HNE-modified histones.	[64]
Omental adipogenesis	Omental adipose tissue of morbidly obese individuals revealed that smaller size of adipocytes, increased adipocytes’ accumulation of 4-HNE-modified proteins and increased adipose tissue macrophage infiltration is associated with impaired adipogenesis.	[33]
Subcutaneous adipogenesis and insulin resistance	The 4-HNE-modified proteins accumulate in subcutaneous adipose tissue of obese individuals, with the highest presence in adipocytes. The 4-HNE affects redox homeostasis and inhibits growth subcutaneous preadipocytes. In addition, 4-HNE affects adipogenic capacity and insulin signaling promoting insulin resistance phenotype.	[34]
Inflammation	Obese individuals have higher amount of circulating 4-HNE. The 4-HNE inhibits miR-29b while it promotes adipokine EST1, resulting in TNF-α upregulation. In obesity, adipokines TNF-α, ETS1, and SP1 are upregulated while miR-29b is downregulated in the subcutaneous white adipose tissue.	[63]
Protein carbonylation	High fat, high sucrose diet induces carbonyl stress and accumulation of 4-HNE adducts and is accompanied with increased GPx4 enzyme in heart and liver.	[66]
Lipolysis	The 4-HNE stimulates lipolysis in adipocytes via upregulation of intracellular cyclic AMP level and increased phosphorylation of protein kinase A, resulting in elevated hormone sensitive lipase. In addition, 4-HNE downregulates AMP-activated protein kinase further supporting lipolysis.	[67]
Adiponectin secretion	HFD-induced obesity is associated with 4-HNE accumulation in adipose tissue and plasma adiponectin reduction. In adipocytes, 4-HNE promotes adiponectin ubiquitination enhancing its degradation via ubiquitin-proteasome system and ultimately resulting in its decreased secretion.	[68]
Protein carbonylation	High fat, high carbohydrate diet downregulates glutathione S-transferase A4 in adipose tissue, allowing 4-HNE carbonylation of proteins including fatty acid binding protein.	[65]
Protein carbonylation	Obesity leads to 4-HNE and 4-hydroxyhexenal carbonylation of nuclear zinc finger proteins.	[69]

Abbreviations: 4-HNE, 4-hydroxynonenal; HFD, high fat diet.

## 4. Surgical Interventions for Obesity and the Impact on Redox Homeostasis

### 4.1. Bariatric Surgery

Medications and conservative methods, which have been preferred in the past, have produced only modest or transient results in the treatment of morbid obesity. In recent years, the surgical approach has become increasingly popular. Surgical treatment of morbid obesity, primarily bariatric surgery, has emerged as an effective treatment option for morbid obesity in recent decades. Standard conservative methods result in long-term success in 4% of patients with morbid obesity, while bariatric procedures have a 70% success rate. The consequences of bariatric surgery have a positive impact not only on the patient’s weight but also on associated chronic diseases such as diabetes, arterial hypertension, respiratory, and sleep disorders [70,71,72]. Because of its impressive results, bariatric surgery became extensively practiced and is today the preferred choice [72].

However, the surgical treatment can be used only after persistent failure of conservative treatment over a long period of time. Currently, indications for bariatric surgery according to the guidelines of International Federation for the Surgery of Obesity and Metabolic Disorders are a BMI greater than 40 kg/m^2^ or a BMI greater than 35 kg/m^2^ and the presence of life-threatening comorbidities. Bariatric surgery is recommended for patients aged 18 to 65 years. The indications for bariatric procedures are still a matter of dispute. Endocrinologists advocate lowering the minimal BMI for bariatric surgery in patients with type 2 diabetes to 32 kg/m^2^. The decision to proceed with surgical treatment must be made jointly by a multidisciplinary team that includes endocrinologists, gastroenterologists, surgeons, psychologists, and nutritionists [70,72].

**Figure 3:**
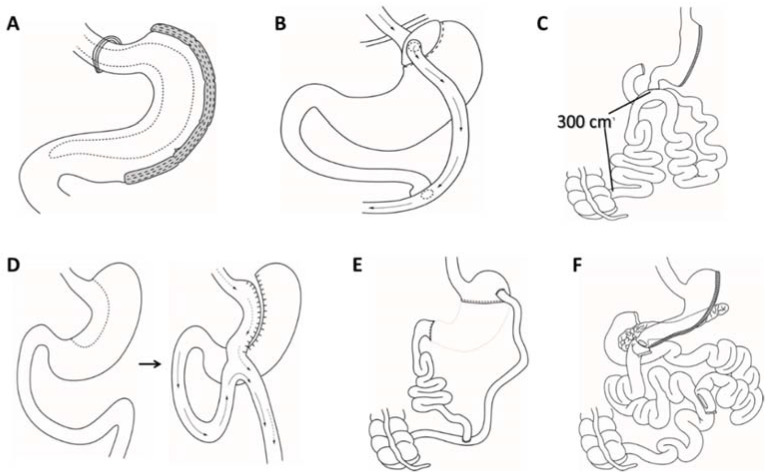
The most common bariatric surgery procedures. (**A**) Vertical sleeve gastrectomy (VSG); (**B**) Roux-en-Y gastric bypass (RYGB); (**C**) Single anastomosis duodeno–ileal bypass with sleeve gastrectomy (SADI-S); (**D**) Mini gastric bypass (MGB); (**E**) Biliopancreatic diversion (BPD); and (**F**) Biliopancreatic diversion with duodenal switch (BPD/DS).

All bariatric procedures performed today can be roughly divided into two categories. One category consists of procedures that result in malabsorption, while the other results in restricted caloric intake. Due to the development of modern techniques, nowadays, most procedures today fall into both categories. Of all the bariatric surgery procedures performed across the globe, patients can be assigned to one of the three procedures: vertical sleeve gastrectomy (VSG), Roux-en-Y gastric bypass (RYGB), and Single anastomosis duodeno–ileal bypass with sleeve gastrectomy (SADI-S) (Figure 3A–C). Other treatments include Mini gastric bypass (MGB) and biliopancreatic diversion with or without a duodenal switch (BPD, BPD/DS) (Figure 3D–F) [70,71,72].

The VSG is the most common bariatric surgery procedure used globally, accounting for around half of all surgeries (Figure 3A) [73]. In VSG, the greater curvature of the stomach, where ghrelin and other gastrointestinal hormones are secreted, is permanently removed by creating a long stomach tube using a cutting/sealing tissue stapler. VSG is generally well tolerated and, in the hands of skilled practitioners, has a very low rate of perioperative complications (<1%), similarly to other bariatric surgeries. Between 50% and 60% of extra body weight is routinely lost after surgery [70,72,74,75].

RYGB is estimated to account for over 40% of all bariatric procedures and is the second most frequent bariatric surgery procedure globally (Figure 3B) [73]. The technique is based primarily on the formation of a small gastric pouch that results in rapid satiety and symptoms of dumping syndrome after the consumption of simple carbohydrates. In RYGB, a small gastric pouch is formed and coupled to the jejunum creating a “Roux” limb (1 to 1.5 m). To re-establish the flow of biliary and pancreatic digestive secretions, the excluded limb of bowel is connected as a jejunojejunostomy, forming a Y-configuration [70]. The remaining distal small bowel is referred to as the common channel. Compared to VSG, RYGB is surgically more challenging, as it features a stomach remnant that stays in place to drain gastric secretions along with two anastomoses or “connections” that are created during surgery. Weight loss after RYGB is comparable to that after VSG, with a reduction in excess body weight up to 50–60% [70]. Postoperative results in terms of weight loss are most convincing in the first years after the procedure, while mortality is five times higher compared to laparoscopic gastric banding [72,76,77,78].

The SADI-S procedure was introduced as a novelty in bariatric surgery in 2007 by Sandzes-Pernaute and collaborators as a modification of BPD/DS (Figure 3C,F). The SADI-S is based on a biliary and pancreatic bypass with the formation of a single anastomosis to simplify the procedure and, consequently, reduce the probability of postoperative complications. It contains two components: a restrictive one, which includes the gastric sleeve restriction, and a malabsorptive one, which includes the duodenal bypass. This procedure is associated with a comparably low complication rate of about 4.8% of all patients. The most frequent complication are loose bowel movements (1.2%). The weight loss reported in literature is around 17.8% of excess body weight in the first trimester after the operation, and increases to up to 100% of excess body weight after two years. The results are comparable to those of RYBG. Although the procedure was introduced as a modification of BPD/DS, only a few studies have compared the results of these two methods [70,72].

The MGB (Figure 3D) is a simplified version of the gastric bypass. The MGB was first performed by Robert Rutledge in 1977 in the USA. The introduction of the procedure faced great backlash (American Society of Metabolic and Bariatric Surgeons AMBS 2000), especially by surgeons from the USA, while the method has been accepted in the rest of the world leading to increasing numbers of performed procedures daily. It is important to note that the philosophy of MGB is based on Billroth II gastric resection. The stomach is resected far from the esophagus, and a long and slim gastric pouch is formed. The gastric remnant is then connected to the small intestine at around 200 cm distal from the Treitz ligament, in cases of greater BMI even more than 250 cm away. Since during the procedure only a loop gastric bypass is rendered, the procedure is technically less challenging and takes less time. Although appearing to be a better and more cost-effective version of the gastric bypass, there is not enough data considering the advantages of this procedure to support this claims since the procedure is still subject of substantial scientific investigation. This procedure is gaining great popularity in Asia, where already MGB now accounts for 20% of all performed bariatric procedures [72,79].

BPD and BPD/DS (Figure 3E,F) are two less prevalent procedures carried out globally (about 1% overall). Similarly to RYGB, both procedures entail extensive rearrangement of the small intestines along with gastric resection, yielding either a smaller stomach pouch or a sleeve-like stomach with BPD or BPD/DS, respectively [70]. In both, BPD and BPD/DS procedure, the distal small ileum (proximal to the colon approximately 100–150 cm) receives the biliopancreatic secretions. With an estimated excess body weight loss of 60–70% [80], BPD and BPD/DS have a greater weight loss efficiency compared to RYGB, but at the expense of greater perioperative morbidity and gastrointestinal adverse effects, as pointed out by some studies [70,81,82].

### 4.2. Mental and Redox Balance Consequences of Bariatric Surgery

Bariatric surgery has numerous benefits for obese patients. In addition to weight loss and reduction in BMI, it improves the metabolic status followed by the decrease in inflammation [83]. The effects of bariatric surgery on metabolic improvement, metabolic diseases, endocrine system and long-term survival have been described in several excellent recent papers [84,85,86,87,88], and are therefore not discussed in this review. The consequences of bariatric surgery on the redox balance as well as a brief overview on the mental consequences are discussed below.

Bariatric surgery improves M2/M1 ratio, favoring M2 polarization of macrophages, as well as it leads to elevated levels of circulating anti-inflammatory IL-10, pointing to resolution of systemic inflammation and reduced oxidative stress [89]. The metabolic function of adipocytes is also improved after procedure [90]. In an animal model, it was suggested that following bariatric surgery renal dysfunction could be alleviated by activating PPARα and by inhibiting of oxidative stress-induced damage [91].

In the last fifteen years, different authors have investigated the oxidative markers after bariatric surgeries, thus generating multiple proofs of reduction in oxidative stress. A study about Swedish adjustable gastric band surgery showed that the postoperative BMI and concentrations of lipid, malondialdehyde (MDA), and oxLDL decreased significantly [92]. Following bariatric surgery, the changes in circulating levels of oxLDL strongly correlate with the change in BMI (%) [93]. Oxidative stress indicators in plasma levels of patients after RYGB procedure showed that MDA was reduced, while GSH and total radical antioxidant parameter were increased, suggesting a drop in oxidative stress and improvement in antioxidant protection [94]. In another study, the authors showed that morbidly obese patients have lower levels of antioxidant enzyme activities and increased lipid peroxidation, which was changed after laparoscopic sleeve gastrectomy during a time course of 1 year. Their results showed the recovery of antioxidant enzyme activities and MDA and F2-isoprostanes (F2-IsoPs) were lowered as well as 8-oxo-7,8- 2′-deoxyguanosine in serum and urine [95]. The decrease in lipid peroxidation markers and protein carbonylation was also reported for morbidly obese women 180 days post RYGB procedure [96]. One study showed that different types of surgeries differently affect oxidative damage markers, revealing that the advanced oxidation protein products (AOPP) were dramatically decreased 6 months post RYGB procedure compared to sleeve gastrectomy [97].

A recent longitudinal study showed that both total antioxidant capacity and total oxidant status are elevated in obese patients regardless of the presence of metabolic syndrome, and following bariatric surgery oxidative stress parameters gradually decreased [98]. The same group of authors showed that obese patients had lower levels of superoxide dismutase (SOD) and reduced GSH but elevated levels of glutathione reductase and uric acid prior to bariatric surgery, as compared to control. Post-surgery SOD increase and uric acid decrease is seen only for the obese patients without metabolic syndrome [71,98]. The oxidative damage to proteins (AGE and AOPP) and lipids (F2-IsoP, 4-HNE) are also elevated in obese individuals, regardless of metabolic syndrome. Although the AGE and AOPP levels decreased after the bariatric surgery, the ratio of reduced to oxidized GSH was still low in the obese individuals with metabolic syndrome, suggesting to consider the antioxidant supplementation in patients undergoing bariatric surgery [71].

Morbid obesity is also associated with oxidative damage to salivary proteins, lipids, and DNA measured by different parameters (4-HNE, F2-IsoP, AOPP, protein carbonyls and 8-hydroxy-D-guanosine) while bariatric treatment decreases the amount of salivary oxidative damage [99].

However, although bariatric surgery improves body redox balance, inadequate diet post surgery can alleviate those effects, as diet affects oxidative stress parameters more than surgery [100]. Types of food consumed after the RYGB procedure were found to correlate with total antioxidant capacity, showing that total antioxidant capacity decreases with higher consumption of ultra-processed foods [101]. Diets high in fat, salt and sugar disturb the redox equilibrium and increase lipid peroxidation [102]. Red meat consumption aggravates this, as the heme iron from red meat promotes lipid peroxidation [103]. Furthermore, although fatty acids are essential macronutrients for humans, the prolonged consumption of food with the shift in the ratio of n-3/n-6 PUFAs in favor of n-6 PUFAs is associated with the development of metabolic syndrome. Therefore, change in diet and lifestyle are essential for obese individuals. The supplementation with antioxidants has also been suggested, to improve the redox balance and ameliorate negative effects of obesity-induced oxidative stress [104,105].

Furthermore, patients undergoing bariatric surgery usually have higher prevalence of various psychiatric disorders, including personality disorder, mood and eating disorders, depression, anxiety and alcohol abuse, compared to healthy control subjects or obese subjects that are not undergoing bariatric surgery [106]. Several studies reported an improvement in these psychological comorbidities after weight loss induced by bariatric surgery [107,108]. For example, two years after surgery, significant alleviation of anxiety and depressive symptoms has been noticed [106,108], following the improvement of quality of life and their body image [109]. However, other studies have reported that weight loss induced by bariatric surgery did not affect anxiety symptoms [107]. Unfortunately, eating disorders persist even after bariatric surgery, which impacts weight loss and weight maintenance [106,109]. However, although bariatric surgery shows improvement in not just medical, but also psychological comorbidities more information is needed on the long-term effects of bariatric surgery on mental health. Additionally, in order to improve mental health, quality of life and weight maintenance, psychotherapy or pharmacotherapy might be suggested for improving postoperative behavior and lifestyle [107].

Taken together, these data highlight the importance to further study the consequences of bariatric surgery, and later lifestyle interventions to improve the surgery selection and/or supplemental therapy addition.

## 5. Conclusions

For a long time, obesity was considered merely a failure of willpower or a character flaw. Recent insights into the pathophysiology of obesity have discovered its complexity, seen in the powerful neural and hormonal control of appetite and energy expenditure. In obesity, redox homeostasis is impaired and shifted towards oxidation affecting macromolecules, leading to formation of 4-HNE and consequently promoting progression of obesity and obesity-associated diseases. The 4-HNE-protein conjugates are difficult to metabolize, and thus, research of proteins modified by 4-HNE in obesity and possible interventions able to augment their formation will allow us to better understand the pathophysiology of obesity-related diseases. Bariatric surgery has positive effects on the treatment of obesity and the medical conditions associated with it. Still, the role of bariatric surgery in the treatment of obesity in minors remains to be evaluated, as well as in less overweight adult individuals. More research on the topic of bariatric surgery and its consequences for patient metabolism and redox homeostasis is critical to understand the underlying mechanisms of obese pathology and to what extent obesity-associated diseases can be alleviated by bariatric surgery.

## Data Availability

Not applicable.
